# Synergistic effects of polyphenols and exercise on obesity: targeting metabolism, muscle function, and adipose tissue remodeling

**DOI:** 10.3389/fnut.2025.1679381

**Published:** 2025-12-01

**Authors:** Xiaowei Lei, Mohammad J. Rezaei

**Affiliations:** 1 Sports Department, Chang’an University, Xi’an, Shaanxi, China; 2 Perelman School of Medicine, University of Pennsylvania, Philadelphia, PA, United States

**Keywords:** polyphenols, exercise, obesity, targeting metabolism, muscle function

## Abstract

Obesity has emerged as an increasingly significant global health dilemma, imposing a considerable strain on healthcare infrastructures globally and markedly heightening the risk of concomitant metabolic disorders. Although regular physical activity and dietary polyphenols are each independently acknowledged for their substantial anti-obesity and metabolic health advantages, the intricate and multifactorial characteristics of obesity necessitate holistic intervention strategies that may surpass the confines of singular therapeutic methodologies. This review amalgamates contemporary scientific literature to investigate the persuasive rationale and empirical evidence supporting the synergistic effects of polyphenols and exercise in addressing obesity. The analysis indicates that their concurrent application can result in improved outcomes across critical physiological domains, encompassing systemic metabolism, muscular functionality, and adipose tissue remodeling. It is noteworthy that integrated interventions have demonstrated a synergistic effect in mitigating weight gain and visceral adiposity, augmenting the browning of white adipose tissue, and enhancing muscle performance as well as recovery. The amalgamation of these two influential modalities presents a promising prospect for the formulation of more effective and comprehensive strategies aimed at the prevention and treatment of obesity, with the potential to address the challenges related to adherence and bioavailability that are often associated with singular interventions.

## Introduction

The global prevalence of obesity has surged dramatically over the past three decades, transforming into a pervasive health crisis with far-reaching societal and economic consequences (1). Characterized by excessive adipose mass, obesity is a multifactorial condition influenced by a complex interplay of lifestyle, behavioral, environmental, and genetic factors, primarily stemming from an imbalance between caloric intake and energy expenditure (2, 3). This pathological state is intimately linked to a cascade of co-morbid metabolic and chronic diseases, including type 2 diabetes, cardiovascular diseases, and metabolic dysfunction-associated steatohepatitis, placing an immense and escalating burden on global healthcare systems (1, 4). A critical feature of obesity’s pathophysiology is the expansion and dysfunction of adipose tissue, which, beyond its role in energy storage, acts as a key endocrine organ. In obesity, this tissue often exhibits chronic low-grade inflammation, contributing to systemic insulin resistance and metabolic derangements (5). This complex pathophysiology underscores the urgent need for multifaceted and effective intervention strategies. This complexity suggests that interventions targeting a single mechanism may be insufficient to comprehensively address the disease, thereby building a strong rationale for exploring combined approaches.

Regular physical activity and structured exercise programs are universally recognized as cornerstone interventions in the management and prevention of obesity and its associated complications (1, 6). Exercise demonstrably contributes to reductions in body weight and fat mass, facilitates the maintenance of these reductions, and profoundly improves overall metabolic fitness (2, 7). Its beneficial effects extend to whole-body metabolism and induce favorable adaptations within key metabolic tissues, including skeletal muscle, adipose tissue, and the liver (1, 8). A particularly compelling aspect of exercise’s impact is its capacity to improve metabolic health and remodel tissues independently of significant weight loss (2, 9). This indicates a profound physiological impact that transcends mere caloric deficit, suggesting that physical activity induces fundamental cellular and tissue-level changes that enhance metabolic resilience. This capacity positions exercise as an indispensable tool in obesity management, capable of yielding significant health benefits even with modest improvements in physical activity levels. Complementing lifestyle interventions, dietary polyphenols, a diverse class of naturally occurring bioactive compounds abundant in plant-based foods, have garnered substantial scientific interest for their recognized health-promoting properties (10, 11). These phytonutrients exhibit considerable anti-obesity potential through a wide array of mechanisms. They can modulate obesity-related digestive enzymes, influence neurohormones and peptides involved in appetite and food intake, and beneficially alter the composition of the gut microbiota (10, 12). Furthermore, polyphenols are potent antioxidants and anti-inflammatory agents, properties crucial for mitigating the chronic low-grade inflammation characteristic of obesity (13). The multi-faceted mechanisms of polyphenols, ranging from gut microbiota modulation to their involvement in immunometabolic pathways and epigenetic reprogramming, indicate they are not simply “antioxidants” but complex biological response modifiers (10, 14). This pleiotropic action, affecting multiple nodes in the obesity pathophysiology network, makes them highly relevant for a complex disease like obesity and positions them as strong candidates for synergistic interactions with other interventions.

Given the independent yet often overlapping mechanisms of action of both exercise and polyphenols, there is a compelling rationale for investigating their combined, synergistic effects in combating obesity. While each intervention is effective on its own, their co-administration could lead to enhanced outcomes by targeting complementary pathways, potentially overcoming individual limitations such as adherence challenges with exercise or bioavailability issues with some polyphenols (15–17). It is important to differentiate here between an additive effect, where the combined benefit equals the sum of the individual interventions (1 + 1 = 2), and a true synergistic effect, where the combination yields a biological response greater than the sum of their individual parts (1 + 1 > 2). The potential for synergy arises from the likelihood that polyphenols can amplify or stabilize the beneficial physiological adaptations induced by exercise, particularly at the molecular and cellular levels. This could result in a more comprehensive and potent strategy for obesity prevention and treatment, achieving outcomes greater than the sum of their individual effects, which is crucial for addressing the chronic and often relapsing nature of obesity. This review aims to comprehensively analyze the existing literature on the combined impact of polyphenols and exercise on metabolism, muscle function, and adipose tissue remodeling.

To synthesize the literature for this review, we conducted a comprehensive search of the PubMed, Scopus, and Web of Science databases for articles published between January 2000 and March 2025. Our search strategy used combinations of the following keywords: (“polyphenol” OR “flavonoid” OR “resveratrol” OR “curcumin” OR “quercetin” OR “EGCG”) AND (“exercise” OR “physical activity” OR “training”) AND (“obesity” OR “adipose tissue” OR “browning” OR “muscle function” OR “metabolism”). We included preclinical (*in vivo* and *in vitro*) studies, clinical trials, meta-analyses, and systematic reviews. Articles were selected for relevance to the independent and combined effects of these interventions, with a focus on synergistic mechanisms. The bibliographies of key articles were also manually scanned for additional relevant publications.

## Polyphenols: independent roles in obesity management

Dietary polyphenols, a diverse group of plant-derived compounds, have emerged as promising agents in the fight against obesity, exhibiting a wide array of mechanisms that modulate metabolic pathways and exert anti-adipogenic effects. These phytonutrients act as potent anti-obesity agents by inhibiting obesity-related digestive enzymes, modulating neurohormones and peptides involved in food intake, and improving the growth of beneficial gut microbes while inhibiting pathogenic ones (10, 18). Specific polyphenols, such as curcumin, ellagic acid, ferulic acid, and quercetin, have been shown to directly inhibit adipocyte production and reduce adverse reactions commonly associated with obesity, including inflammation, insulin resistance, and gut microflora imbalance (19, 20). Polyphenols can influence thermogenesis by modulating adipose tissue phenotypes, promoting the conversion of energy-storing white adipose tissue (WAT) into thermogenic brown adipose tissue (BAT) or “beige” adipose tissue, a process known as WAT browning (21). For instance, ellagic acid facilitates this transformation by inhibiting WAT maintenance genes like Zfp423 and Aldh1a1 and increasing the expression of beige markers such as CD137 and TMEM26. Curcumin can boost BAT activity by regulating bile acid metabolism, which increases levels of Takeda G-protein-coupled receptor 5’s ligands, deoxycholic acid and lithocholic acid. Quercetin has also been observed to elevate Uncoupling Protein 1 (Ucp1) levels and thermogenesis-related gene expression (19, 22, 23).

Polyphenols also inhibit fat formation through precise regulation of signal pathways and transcription factors. They can influence the Rb protein signaling pathway, which is crucial in early fat cell development. Ellagic acid and curcumin, for example, significantly inhibit hormone-induced lipogenesis and block the G1/S phase shift, thereby impeding the terminal differentiation of pre-adipocytes and lipid accumulation. The P38 mitogen-activated protein kinase (P38 MAPK) pathway, which is amplified during adipocyte maturation, can be modulated by curcumin to reduce triglyceride buildup. Curcumin further suppresses adipogenesis by stimulating the conventional Wnt signaling pathway, which impedes the transcriptional activities of key adipocyte differentiation controllers like PPAR-*γ* and C/EBP-*α*. Ellagic acid also suppresses histone arginine methyltransferase 4 (CARM1), a protein vital for adipogenesis (19, 24, 25). Beyond formation, polyphenols regulate fat metabolism. Ellagic acid has been shown to catalyze lipase activity, leading to a marked elevation in proteins linked to lipid metabolism, including triglyceride lipase and phosphorylated hormone-sensitive lipase. Many polyphenols, including ellagic acid and quercetin, activate the AMP-activated protein kinase (AMPK) pathway, a master regulator of *in vivo* lipid synthesis, fatty acid catabolism, and oxidation. AMPK activation promotes *β*-oxidation and inhibits lipogenesis and triglyceride resynthesis (13, 26). Furthermore, polyphenols play a significant role in alleviating insulin resistance. They modulate the PI3K/AKT signaling pathway (e.g., quercetin, ellagic acid, curcumin, ferulic acid) and activate the AMPK pathway (e.g., quercetin, ellagic acid), thereby enhancing glucose uptake in skeletal muscle and fat tissues and improving overall insulin sensitivity and glucose metabolism (19, 27). Their anti-inflammatory properties are also critical, as they can inhibit inflammatory factors such as TNF-*α*, IL-6, and IL-1 (e.g., quercetin, ellagic acid, curcumin, ferulic acid) and suppress NF-κB through AMPK pathway regulation, which is crucial for mitigating the chronic low-grade inflammation associated with obesity (19, 28).

**Figure 1:**
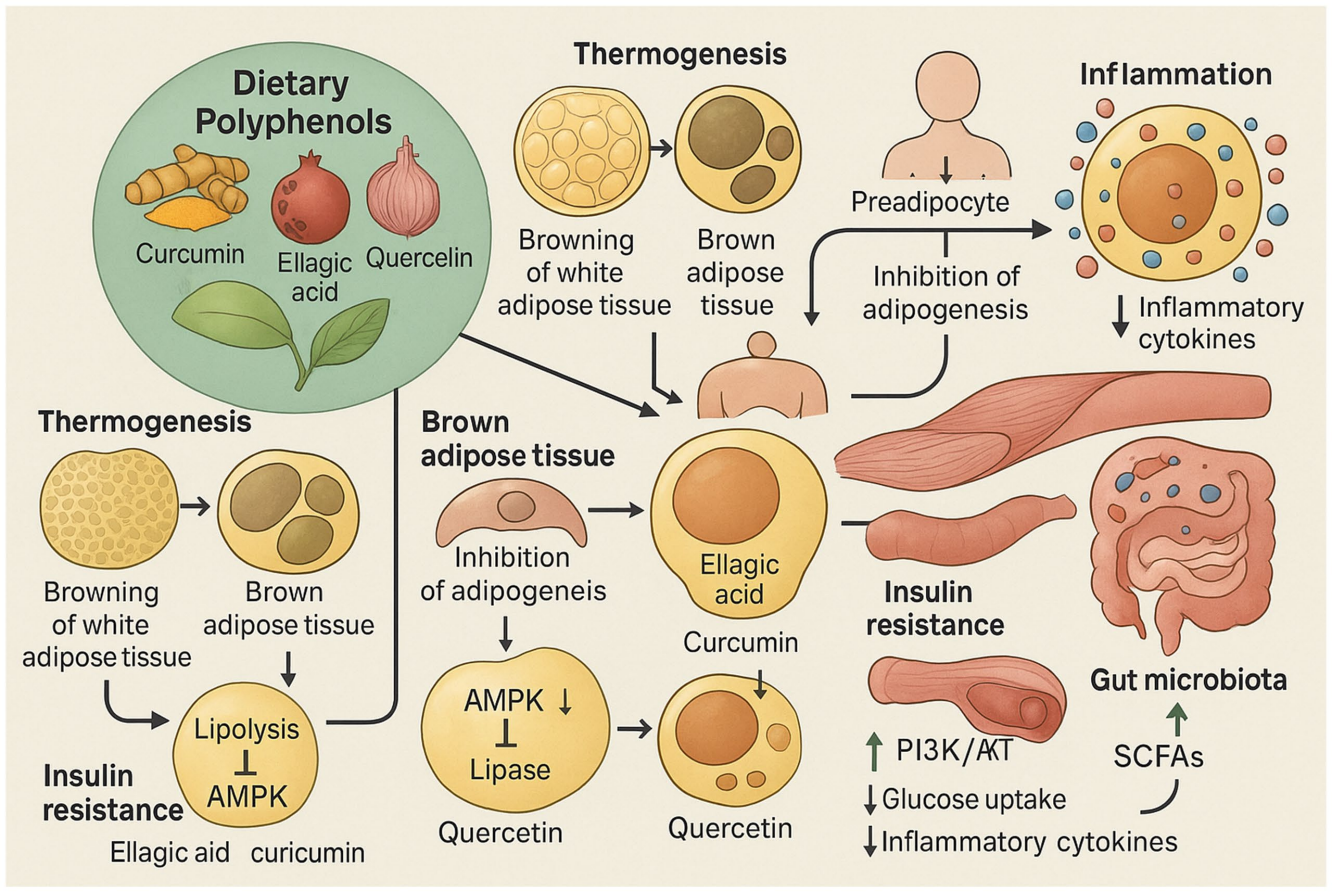
This diagram illustrates the anti-obesity mechanisms of dietary polyphenols such as curcumin, ellagic acid, and quercetin. These compounds promote thermogenesis by browning white adipose tissue, inhibit adipogenesis by blocking fat cell differentiation, enhance lipid metabolism via AMPK activation, and improve insulin sensitivity through PI3K/AKT and AMPK pathways. They also reduce inflammation by suppressing cytokines and modulate gut microbiota to increase beneficial short-chain fatty acids (SCFAs), contributing to metabolic balance and obesity prevention.

Finally, polyphenols profoundly influence the gut microbiome, altering its composition, ratio, and variety. Ellagic acid, curcumin, quercetin, and ferulic acid have all been shown to mitigate obesity and metabolic issues by adjusting intestinal flora, boosting microbiota diversity, reducing Firmicutes, and promoting beneficial short-chain fatty acid-producing bacteria (19). The extensive array of molecular targets and signaling pathways modulated by polyphenols indicates their profound influence on energy balance and metabolic homeostasis. This multi-target approach is critical for addressing the systemic dysregulation seen in obesity; however, it also highlights the inherent complexity in determining optimal dosages and specific polyphenol efficacy, as different compounds may have varying affinities and potencies for these diverse targets. For example, recent mechanistic studies show that quercetin can ameliorate obesity and inflammation by modulating the gut microbiota to promote the production of metabolites like indole-3-propionic acid (IPA), which in turn enhances intestinal barrier integrity and suppresses chronic inflammation (29) (Figure 1).

### Influence on muscle health and performance

Polyphenols are increasingly recognized for their significant role in enhancing muscle health and performance, particularly in alleviating post-exercise muscle damage and reducing fatigue. Their potent antioxidant and anti-inflammatory properties are critical for combating oxidative stress and inflammation, two factors that intensify during vigorous physical activity and contribute to muscle soreness and damage (30, 31). Quercetin, a prominent flavonoid, has particularly emerged as an effective agent for promoting muscle recovery and enhancing exercise performance. It reduces exercise-induced oxidative stress, evidenced by lower malondialdehyde (MDA) levels and increased total antioxidant capacity (TAC) and superoxide dismutase (SOD) activities during recovery. Simultaneously, it mitigates pro-inflammation by reducing levels of inflammatory cytokines like interleukin-6 (IL-6) and muscle damage markers such as creatine kinase (CK) and lactate dehydrogenase (LDH) (30, 32, 33).

Beyond damage mitigation, quercetin enhances neuromuscular performance, improving the torque-velocity curve for knee extensors and reducing the decrease in maximal voluntary isometric contraction (MVIC) following resistance exercise. It also increases the rate of torque development and neuromuscular efficiency. Quercetin supplementation has been observed to extend high-intensity cycling time to exhaustion, directly improving exercise performance. Its ability to accelerate muscle recovery is evident in reduced strength loss and decreased levels of muscle damage markers (30, 34, 35). Furthermore, quercetin modulates growth factors, increasing insulin-like growth Factor-I (IGF-I) levels, which are crucial for muscle repair and regeneration. It also mitigates mitochondrial oxidative stress by preventing glutathione depletion and reducing reactive oxygen species (ROS) production, thereby protecting against mitochondrial dysfunction (30, 36).

Various other polyphenols contribute to muscle health by attenuating muscle atrophy and enhancing anabolism. This is achieved through multiple mechanisms, including activating protein synthesis pathways like Akt/mTOR, inhibiting protein degradation by suppressing E3 ubiquitin ligases (e.g., atrogin-1, MuRF-1), enhancing mitochondrial function and biogenesis (via PGC-1α and SIRT1), and stimulating myogenesis for muscle cell growth and differentiation (37–39). Examples include gallic acid, ellagic acid, urolithin A and B, ferulic acid, epicatechin, epigallocatechin gallate (EGCG), hesperidin, apigenin, luteolin, genistein, daidzein, resveratrol, and curcumin, all demonstrating anti-atrophic effects in various models (37, 40). Polyphenols’ dual action in both protecting against exercise-induced damage and directly promoting muscle anabolism and mitigating atrophy positions them as comprehensive agents for muscle health. This suggests that they can optimize the adaptive responses to physical activity, which is a critical component of obesity management, by allowing for more effective training and recovery.

### Impact on adipose tissue remodeling

Polyphenol supplementation has a notable impact on adipose tissue remodeling, leading to significant decreases in markers of central adiposity, including percentage of body fat, fat mass, and waist circumference. Furthermore, it effectively reduces visceral adiposity, specifically visceral adipose tissue, which is closely linked to cardiometabolic risk (13, 41). This is strongly supported by recent quantitative evidence. A large 2023 systematic review and meta-analysis of 59 RCTs (3,802 participants) found that GTE supplementation significantly reduced body mass (Weighted Mean Difference [WMD]: −0.66 kg; 95% CI: −1.04, −0.28), BMI (WMD: −0.25 kg/m^2^; 95% CI: −0.42, −0.08), and body fat percentage (WMD: −0.52%; 95% CI: −0.84, −0.19) (42). The same study confirmed GTE’s role in mitigating oxidative stress, finding a significant reduction in malondialdehyde (MDA) and an increase in total antioxidant capacity (TAC) The mechanisms underlying these anti-adipogenic effects are complex and involve multiple pathways. Polyphenols activate lipid turnover pathways and the AMP-activated protein kinase (AMPK) pathway, which plays a central role in energy metabolism. They also suppress key transcription factors involved in lipid synthesis within adipose tissue, such as SREBPs, PPAR-*γ*, and C/EBP-*α*, as well as crucial enzymes in these synthetic processes (13, 43, 44). Beyond direct lipid metabolism, polyphenols modulate the immune system within adipose tissue, which is often characterized by chronic low-grade inflammation in obesity (45). They exert immunomodulatory actions through a mitochondria-centered multi-modal approach, activating nutrient sensing via stress-response pathways and interfering with the assembly of the NLR family pyrin domain containing 3 (NLRP3) inflammasome at endoplasmic reticulum-mitochondria contact sites. This inhibition of NLRP3 activation, coupled with improved mitochondrial biogenesis and autophagosome-lysosome fusion, contributes to alleviating inflammation (45–47). Moreover, polyphenols impact chromatin remodeling and coordinate both epigenetic and metabolic reprogramming, offering new insights into their multifaceted nature beyond well-documented antioxidant properties (45, 48). Polyphenols directly influence adipose tissue morphology and function beyond simple fat mass reduction, engaging in complex immunometabolic and epigenetic modulation. This indicates a sophisticated regulatory role that could complement exercise-induced adipose tissue remodeling, leading to a healthier and more metabolically active fat phenotype (Table 1).

**Table 1:** Key polyphenols and their independent anti-obesity mechanisms.

Polyphenol compound	Primary source(s)	Target area(s)	Key mechanism(s) of action	Reference
Curcumin	Turmeric	Metabolism, muscle, adipose	Inhibits digestive enzymes, modulates neurohormones, improves gut microbes, boosts BAT activity, inhibits fat formation (Wnt, Rb), alleviates insulin resistance (PI3K/AKT, STAT3), anti-inflammatory (COX-1, COX-2, MMP), attenuates muscle atrophy (p38 MAPK, ubiquitination), mitochondrial protection.	(5)
Ellagic acid	Berries, pomegranate	Metabolism, muscle, adipose	Inhibits adipocyte production, reduces inflammation, improves gut microflora, promotes WAT browning, inhibits fat formation (Rb, PPARγ, C/EBP-α, CARM1), catalyzes lipase, activates AMPK, alleviates insulin resistance (PI3K/AKT, AMPK), suppresses NF-κB, protects muscle (oxidative stress, inflammation).	(13)
Ferulic acid	Grains, fruits	Metabolism, muscle, adipose	Reduces inflammation, improves gut microflora, alleviates insulin resistance (PI3K/AKT/GLUT4, FOXO1), reduces inflammatory markers, promotes muscle growth (MyHC, Sirt1, PGC-1α, MEF2C, AMPK).	(19)
Quercetin	Fruits, vegetables, tea	Metabolism, muscle	Reduces inflammation, insulin resistance, gut microflora imbalance, elevates Ucp1, enhances thermogenesis, alleviates insulin resistance (PI3K/AKT, AMPK, GLUT4), anti-inflammatory (IL-10, TNF-α, IL-1), modulates gut microbiome, reduces oxidative stress, enhances neuromuscular performance, improves exercise performance, accelerates muscle recovery, modulates growth factors (IGF-I), mitigates mitochondrial oxidative stress, attenuates muscle atrophy (MuRF-1, Atrogin-1, NF-κB inhibition, HO-1 activation).	(19)
Resveratrol	Grapes, red wine, peanuts	Metabolism, muscle	Modulates signaling pathways (AMPK, PPARγ, SIRT1), enhances energy expenditure, stimulates lipolysis, fatty acid *β*-oxidation, reduces inflammation, activates SIRT1-PGC-1α-NRF-1-TFAM pathway, attenuates muscle atrophy (ubiquitin ligases, oxidative stress, antiapoptotic), acts as exercise mimetic.	(5)
EGCG	Green tea	Metabolism, muscle	Enhances cellular ROS production, activates AMPK, suppresses adipogenic enzymes/transcription factors, reduces inflammation, suppresses muscle atrophy (ubiquitin-proteasome, myostatin, atrogin-1, MuRF-1, Akt, FoxO1a, NF-κB), promotes mitochondrial biogenesis.	(5)
Gallic acid	Tea, berries	Muscle	Improves mitochondrial functions (SIRT-1, PGC-1α, Nrf1, TFAM), promotes mitochondrial biogenesis, oxidative phosphorylation, autophagy/mitophagy, increases muscle differentiation (myogenin, Myf5, MyoD).	(37)
Urolithin A/B	Pomegranate metabolites	Muscle	Stimulates muscle function, exercise capacity (autophagy, mitophagy), preserves mitochondrial function, activates protein synthesis (androgen receptor, mTOR), inhibits protein degradation (ubiquitin ligases, myostatin).	(37)
Apigenin	Parsley, celery	Muscle	Prevents aging-induced muscle loss, increases fiber size, reduces oxidative stress, inhibits mitophagy/autophagy/apoptosis, enhances mitochondrial function/biogenesis (AMPK activation), ameliorates obesity-induced atrophy, suppresses inflammatory cytokines.	(37)

While polyphenols provide a powerful biochemical intervention, they represent only one half of the synergistic strategy this review explores. We now turn to the other foundational component of this approach: exercise, which induces profound physiological adaptations that complement the actions of polyphenols.

## Exercise: a foundational strategy against obesity

Exercise stands as a foundational strategy in the comprehensive management of obesity, inducing profound systemic metabolic adaptations that extend beyond mere caloric expenditure. It is a well-established measure to prevent or mitigate the adverse consequences of obesity, offering numerous beneficial effects on whole-body metabolism and inducing favorable adaptations within key metabolic tissues, including muscle, adipose tissue, and liver (1, 49). Regular physical activity consistently contributes to reductions in body weight and fat, facilitates the long-term maintenance of these reductions, and significantly improves metabolic fitness in individuals with obesity (2, 50). The efficacy of exercise is so profound that it has the potential to alleviate the health consequences of obesity even in the absence of significant weight loss, underscoring its direct physiological impact on energy homeostasis and metabolic health (2, 51). This capacity to induce systemic metabolic improvements and tissue adaptations independently of changes in body mass highlights its fundamental role in improving metabolic resilience. Different modalities of exercise contribute distinctly to these benefits. Endurance training appears to be particularly effective in its overall impact on body weight, fat loss, and metabolic fitness. However, resistance training and high-intensity interval training (HIIT) also play crucial roles in the effectiveness of exercise interventions for obesity management (2, 52). At a mechanistic level, a single bout of exercise acutely stimulates adipose tissue blood flow and fat mobilization, ensuring that fatty acids are delivered to skeletal muscles at a rate well-matched to metabolic requirements (9). Following an exercise session, there is a period during which fatty acids are redirected away from adipose tissue towards other tissues, such as skeletal muscle, thereby reducing dietary fat storage in adipose tissue. With chronic exercise training, there are sustained changes in adipose tissue physiology, notably an enhanced ability to mobilize fat during acute exercise (9, 53).

### Enhancement of muscle mass, strength, and function

Exercise plays a critical role in enhancing muscle mass, strength, and overall physical performance. In older adults, including those with sarcopenia, exercise has a substantial positive impact on physical performance and a medium effect on muscle strength (54). However, its effect on muscle mass can be inconsistent, with some studies showing no significant change. This variability in muscle mass response suggests a potential area where complementary interventions could provide additional benefit (54, 55). Resistance training frequency is a key variable influencing muscle hypertrophy. Current evidence indicates that training muscle groups at least twice a week promotes superior hypertrophic outcomes compared to once a week, suggesting that higher frequency is associated with greater muscle growth (56). Beyond direct muscular adaptations, exercise triggers the release of neurotrophic factors, endorphins, and various neurotransmitters that are crucial for maintaining brain health and regulating emotions. This neurobiological impact translates into improved cerebral blood flow and oxygen supply, which can indirectly support motivation and adherence to physical activity regimens (57–59). While exercise consistently improves muscle strength and physical performance, its variable effect on muscle mass indicates a potential gap. This suggests that polyphenols, with their documented anti-atrophic and pro-anabolic effects, could provide the necessary support to achieve more consistent improvements in muscle mass, leading to a more comprehensive enhancement of muscle health within the context of obesity management.

### Remodeling of adipose tissue physiology

**Figure 2:**
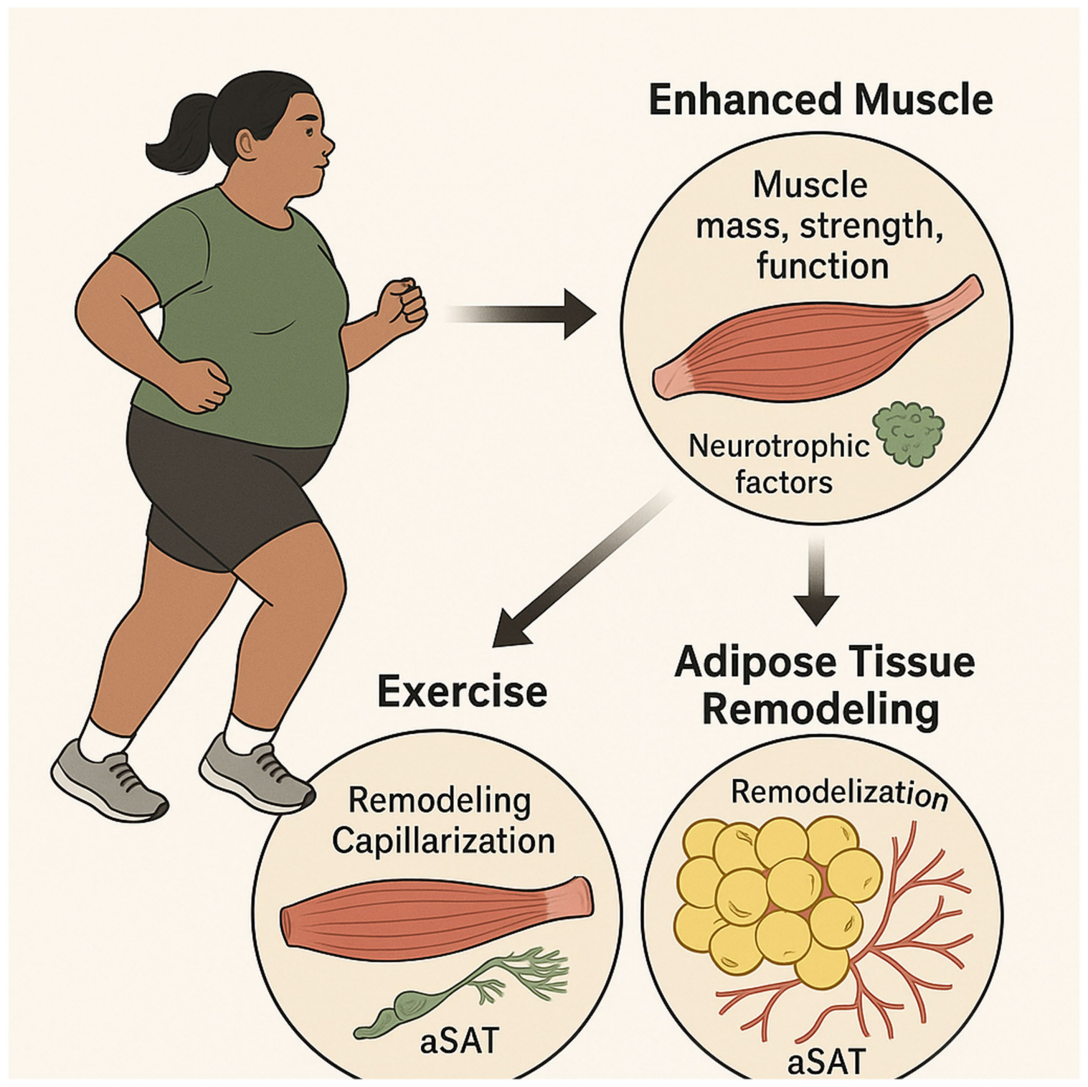
This illustration shows how exercise combats obesity by enhancing muscle mass, strength, and neurotrophic support, while remodeling adipose tissue (aSAT) through improved capillarization and reduced adipocyte size. These adaptations improve metabolic health, even without significant weight loss.

Exercise training profoundly remodels subcutaneous adipose tissue (aSAT) in adults with obesity, remarkably, even without concomitant weight loss. This remodeling involves significant changes in aSAT morphology, including a reduction in fat cell size, an increase in Collagen type 5a3, and an increase in capillary density (60). These structural adaptations are crucial for improving metabolic health, as hypertrophic adipocytes and reduced capillarization are tightly linked to insulin resistance and impaired fat storage capacity in obesity (60, 61). The underlying mechanisms involve alterations in the protein abundance of factors that regulate aSAT remodeling. For instance, exercise training reduces matrix metallopeptidase 9 (MMP9) and increases angiopoietin-2, both key proteins in extracellular matrix remodeling and angiogenesis (60, 62). Furthermore, a 2025 systematic review highlights that exercise interventions are a key strategy to ameliorate the negative effects of adipose tissue aging, in part by enhancing the browning of white adipose tissue and suppressing chronic inflammation (63). Furthermore, exercise increases the protein abundance of factors that regulate lipid metabolism, such as hormone-sensitive lipase (HSL) and fatty acid translocase (FAT/CD36), and elevates mitochondrial markers like COX-IV, indicative of increased mitochondrial density and oxidative capacity. While some of these changes, particularly in the mitogen-activated protein kinase (MAPK) pathway, can be transient, others like the increase in COX-IV persist for several days after the last exercise session (60, 64, 65). Exercise training is also known to reduce the weight of both subcutaneous and visceral white adipose tissue (WAT) in experimental animals and humans, with a dose–response relationship observed between the amount of training and the extent of WAT mass reduction (66, 67). This reduction can involve either a decrease in adipocyte numbers, particularly if training is initiated early in life, or primarily a reduction in adipocyte size, achieved largely by suppressing adipocyte differentiation rather than inducing apoptosis of mature adipocytes (66, 68). Exercise’s direct remodeling effects on adipose tissue, including favorable changes in adipocyte morphology and capillarization, are crucial for improving metabolic health beyond simple fat mass reduction (62, 69). This tissue-specific adaptation provides a fertile ground for synergistic interaction with polyphenols, which also target adipose tissue remodeling and inflammation, potentially leading to more profound and sustained benefits (70, 71) (Figure 2).

### Combined and synergistic effects of polyphenols and exercise in combating obesity

The combined application of polyphenols and exercise represents a promising frontier in obesity management, with emerging evidence pointing towards synergistic effects that surpass the benefits of either intervention alone. This combined approach leverages the distinct yet complementary mechanisms of both modalities to exert a more potent and comprehensive impact on systemic metabolism, muscle function, and adipose tissue remodeling (72, 73).

### Combined impact on whole-body metabolism and energy homeostasis

One of the most compelling demonstrations of synergy lies in the combined impact on whole-body metabolism, particularly concerning adipose tissue dynamics. The combination of aerobic exercise and resveratrol (RES) has been shown to synergistically suppress weight gain and visceral fat accumulation. This profound effect is attributed to an enhanced white adipose tissue (WAT) browning, evidenced by the upregulation of uncoupling protein 1 (UCP1) and cell death-inducing DFFA-like effector A (CIDEA) expression (15, 74, 75). Mechanistically, this combined intervention promotes WAT browning and lipolysis by enhancing mitochondrial biogenesis and stimulating mitochondrial thermogenesis through the modulation of the SIRT1-PGC-1α-NRF-1-TFAM pathway. Resveratrol itself is known to activate SIRT1, which in turn activates PGC-1α, a key regulator of mitochondrial biogenesis, a pathway also strongly influenced by exercise (15, 76, 77). This convergence on a critical energy-expending pathway highlights a true synergistic interaction. Similarly, a low dose of curcumin (1.5 mg/kg, daily) combined with exercise synergistically induced beige adipocyte formation in inguinal white adipose tissue (iWAT) in mice (78). This effect was associated with an elevation of interleukin-6 (IL-6) levels, suggesting that exercise, when combined with curcumin, enhances biological activity and reduces the required curcumin dose to achieve beneficial metabolic outcomes (78, 79).

Furthermore, the combined effects of green tea catechins (GTCs), particularly epigallocatechin gallate (EGCG), with exercise have demonstrated beneficial outcomes in human trials. GTCs combined with exercise have shown positive effects on abdominal fat loss in overweight and obese adults, leading to improved body weight, BMI, and waist circumference (80–82). Green tea consumption combined with resistance training has also been reported to decrease body fat, waist circumference, and triglyceride levels, while simultaneously increasing lean body mass and muscle strength (80, 83). However, it is important to acknowledge that synergy is not a universal phenomenon across all physiological outcomes and specific combinations. For instance, one study investigating grape polyphenols and exercise training in obese insulin-resistant rats found that while both interventions independently prevented pathological cardiac hypertrophy, their combination showed an additive, but not synergistic, benefit compared to exercise alone (84, 85). This finding suggests that the effects of polyphenol supplementation can be physical-activity-status-specific, potentially offering more protection in sedentary obese rats than in exercised ones (84, 86). The C. However, the lack of universal synergy in all contexts indicates that the specific polyphenol, the exercise modality, and the target physiological outcome are critical determinants of the presence and magnitude of synergistic effects, necessitating precise research designs for future investigations (87, 88).

### Augmenting muscle function, recovery, and anabolism

**Figure 3:**
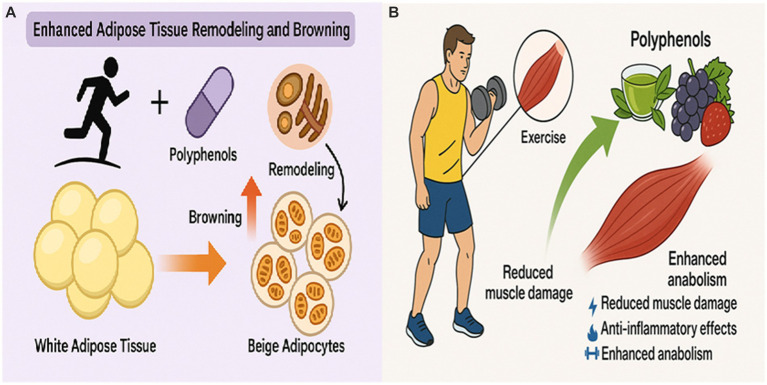
**(A)** Enhanced adipose tissue remodeling and browning represent a potent synergistic outcome of combining polyphenol supplementation with aerobic exercise. This dual intervention promotes the conversion of white adipose tissue (WAT) into metabolically active beige adipocytes, increasing energy expenditure through non-shivering thermogenesis. While both exercise and polyphenols independently reduce WAT mass and improve adipokine profiles, their combination enhances functional tissue transformation more effectively than either alone. This shift from energy storage to energy dissipation contributes significantly to improved systemic metabolic health in obesity management. **(B)** This illustration shows how polyphenols enhance the benefits of exercise by reducing muscle damage, lowering inflammation, and promoting anabolism. Together, they support faster recovery, greater muscle strength, and improved adaptation to physical training.

The synergistic relationship between polyphenols and exercise extends significantly to muscle function, recovery, and anabolism. Exercise, while beneficial for muscle adaptation, can induce oxidative stress and inflammation, leading to muscle damage and fatigue (30, 37, 89). Polyphenols, with their potent antioxidant and anti-inflammatory properties, play a crucial role in mitigating these negative consequences, thereby allowing for more effective adaptive responses and recovery (30, 90). Quercetin, for example, has been shown to alleviate post-exercise muscle damage by actively combating the oxidative stress and inflammation intensified during vigorous physical activity. It promotes muscle recovery and enhances exercise performance through multiple mechanisms, including modulating inflammatory pathways, accelerating muscle repair processes, and enhancing mitochondrial function (30). Quercetin supplementation specifically reduces muscle weakness and electromyographic alterations during eccentric exercise, indicating a protective effect against exercise-induced myofibrillar disruption and sarcolemmal action potential propagation impairment (91). Similarly, a 2024 systematic review and meta-analysis on polyphenol-rich berry supplementation found that while effects on performance were not conclusive, berry consumption showed a clear trend towards attenuating inflammation and oxidative stress markers post-exercise (92). While exercise independently improves muscle strength and physical performance, as evidenced in studies on sarcopenia where it significantly impacts physical performance and strength, polyphenols can further augment these benefits and mitigate damage (54, 93). The combined effects of polyphenols and exercise on muscle health also extend to neuroprotection and anti-inflammatory effects, which can indirectly support sustained physical activity and enhance recovery by reducing systemic inflammation and improving overall well-being (37, 57). Furthermore, specific polyphenols like EGCG, when combined with resistance training, have been shown to increase lean body mass and muscle strength, indicating a direct synergistic effect on muscle anabolism (80). The synergy in muscle function appears to stem from polyphenols’ ability to mitigate the negative consequences of exercise (e.g., oxidative stress, inflammation, damage) while simultaneously enhancing the adaptive responses (e.g., mitochondrial biogenesis, anabolism). This dual protective and potentiating role allows for more effective training adaptations and recovery, which is vital for sustained physical activity in obesity management and for optimizing the metabolic benefits of lean mass (31, 94, 95) (Figure 3A; Table 2).

**Table 2:** Documented synergistic effects of polyphenol-exercise combinations on obesity parameters.

Polyphenol(s) used	Exercise type/protocol	Observed synergistic outcome(s)	Proposed synergistic mechanism(s)	Notes/context	Ref
Resveratrol	Aerobic exercise	Suppressed weight gain, reduced visceral fat accumulation, enhanced WAT browning	Enhanced mitochondrial biogenesis, stimulated mitochondrial thermogenesis via SIRT1-PGC-1α-NRF-1-TFAM pathway modulation	Animal study (obese rats)	(15)
Curcumin	Exercise	Synergistic beige adipocyte formation in iWAT	Elevation of interleukin-6 (IL-6) levels, enhanced biological activity, reduced required curcumin dose	Animal study (mice), low dose curcumin	(78)
Green tea catechins (GTCs), EGCG	Exercise, Resistance training	Abdominal fat loss, improved body weight, BMI, waist circumference, increased lean body mass, muscle strength	Enhanced cellular ROS production, AMPK activation, suppression of adipogenic enzymes/transcription factors	Human clinical trials, some inconsistencies noted	(80)
Quercetin	Eccentric exercise	Attenuated muscle weakness, reduced electromyographic alterations	Antioxidant and anti-inflammatory properties, protection against myofibrillar disruption	Human study	(132)
Grape polyphenols	Exercise training	*No additional benefit* for cardiac hypertrophy	Physical-activity-status-specific effects; potentially more protective in sedentary obese rats	Animal study (obese insulin-resistant rats), highlights context-dependency	(84)
Curcumin	Exercise training + Calorie Restriction	Reduced body weight regain post-weight loss, suppressed appetite (indirect effect)	Inhibition of glucocorticoid action and inflammation (proposed)	Animal study (rats), indirect effect on appetite noted	(133)

### Enhanced adipose tissue remodeling and browning

The most direct and potent synergistic effect observed in the context of obesity is the enhanced remodeling and browning of adipose tissue. As previously highlighted, the combination of resveratrol and aerobic exercise synergistically promotes white adipose tissue (WAT) browning and lipolysis (15, 96). This transformation of energy-storing WAT into metabolically active beige adipocytes, which are characterized by increased energy expenditure through non-shivering thermogenesis, represents a highly desirable outcome in obesity that is often difficult to achieve with single interventions (15, 97). Similarly, low-dose curcumin, when combined with exercise, synergistically induces beige adipocyte formation, further underscoring the potential of these combined interventions to functionally transform adipose tissue (78, 98). Both exercise training and polyphenol intake independently contribute to a reduction in white adipose tissue (WAT) mass and beneficially influence adipokine expression, which are crucial for systemic metabolic health (66, 99). While polyphenols alone can downregulate pathways contributing to adipogenesis, cell cycle, and apoptosis in subcutaneous adipose tissue, exercise also independently remodels subcutaneous adipose tissue (aSAT) morphology, leading to reduced fat cell size and increased capillary density even without weight loss (41, 60, 100). The synergy likely lies in their combined ability to promote a healthier, more metabolically active adipose tissue phenotype. This represents a functional remodeling of adipose tissue towards an energy-expending phenotype rather than just a quantitative reduction in fat mass, thereby impacting systemic metabolism more profoundly (Figure 3B; Table 2).

## Shared molecular pathways and mechanisms of interaction

The compelling evidence for synergy between polyphenols and exercise in combating obesity stems from their convergence on several fundamental molecular pathways and physiological mechanisms that are critical for metabolic health (73). This multi-level interaction suggests that polyphenols can act as “metabolic sensitizers” or “performance enhancers” for exercise, optimizing the body’s adaptive responses and potentially overcoming metabolic inflexibility characteristic of obesity (101). One of the most prominent shared pathways is the activation of AMP-activated protein kinase (AMPK). Numerous polyphenols, including curcumin, ellagic acid, quercetin, resveratrol, and EGCG, are known to activate AMPK (5, 102). Concurrently, exercise is a potent physiological activator of AMPK (15, 103). As a master regulator of energy homeostasis, AMPK promotes fatty acid catabolism and oxidation, enhances glucose uptake, and inhibits lipogenesis (19, 104). The concurrent activation of AMPK by both interventions suggests a convergent mechanism that leads to a stronger, more sustained metabolic signal, thereby improving overall metabolic fitness (105). Another crucial point of convergence is the SIRT1-PGC-1α pathway. Resveratrol, for instance, activates SIRT1, which in turn activates PGC-1α, a key regulator of mitochondrial biogenesis (5, 106). Exercise also strongly induces PGC-1α expression (15, 107). The synergistic effect of exercise and resveratrol on white adipose tissue browning is explicitly linked to the enhanced modulation of the SIRT1-PGC-1α-NRF-1-TFAM pathway, leading to increased mitochondrial biogenesis and thermogenesis (15). This indicates that polyphenols can amplify or fine-tune the exercise-induced mitochondrial adaptations, leading to more profound metabolic shifts. A new dimension to this molecular synergy involves the regulation of autophagy. A 2025 review highlights that both polyphenols (like resveratrol and curcumin) and exercise are potent modulators of autophagy, a critical cellular recycling process. The combined interventions may synergistically enhance autophagic flux through shared pathways like AMPK/mTOR and SIRT1/FOXO, which is crucial for clearing damaged mitochondria and proteins, thereby improving metabolic health and cellular adaptation (108).

**Figure 4:**
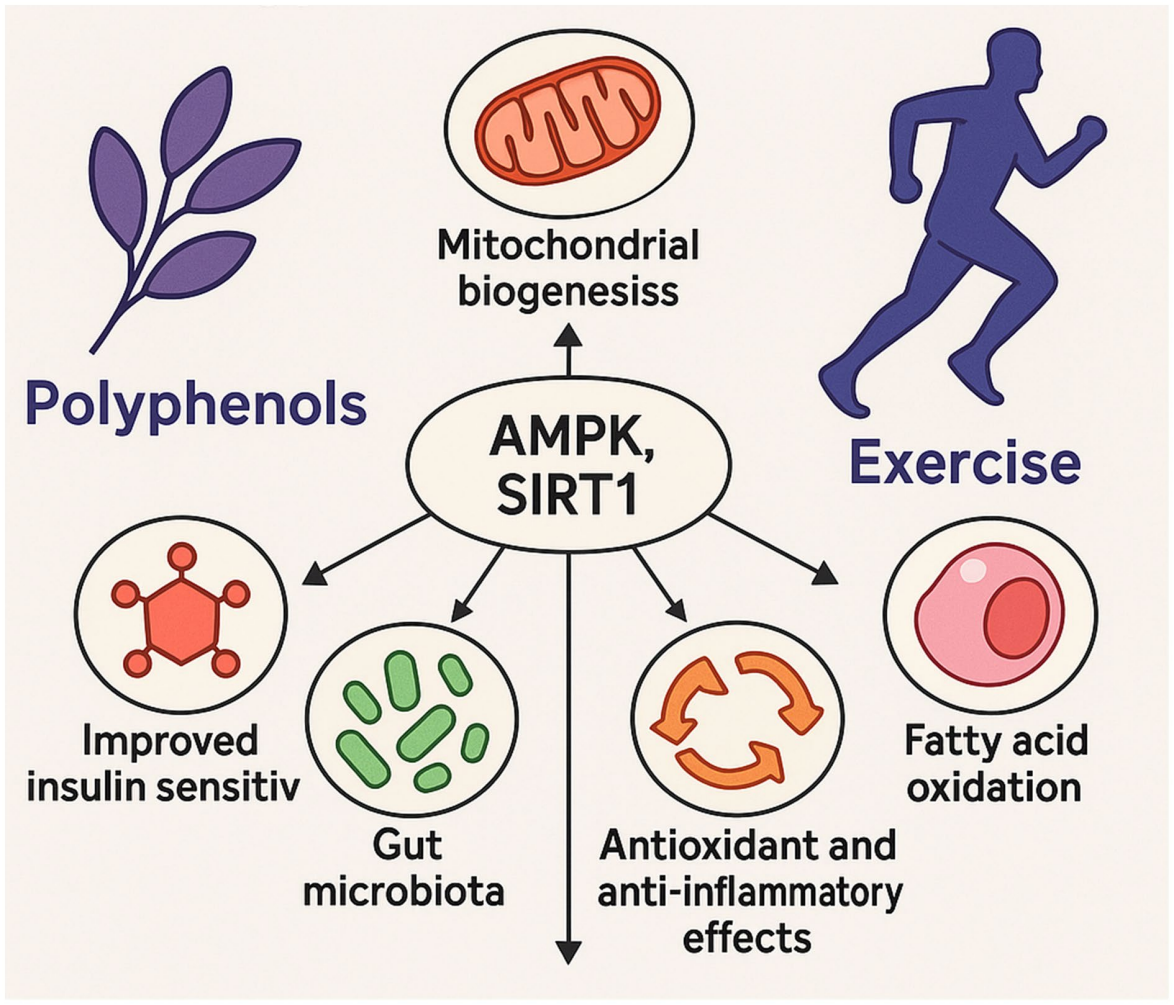
This illustration highlights the synergistic effects of polyphenols and exercise through shared activation of AMPK and SIRT1 pathways. Together, they enhance mitochondrial biogenesis, fatty acid oxidation, antioxidant and anti-inflammatory defenses, gut microbiota composition, and insulin sensitivity—key mechanisms in combating obesity and improving metabolic health.

Both polyphenols and exercise exert potent antioxidant and anti-inflammatory effects, representing a crucial shared mechanism for alleviating the chronic low-grade inflammation associated with obesity (5, 109). While exercise can induce a transient increase in oxidative stress and inflammation, polyphenols effectively mitigate these negative effects, allowing for better adaptation, recovery, and reduced systemic inflammatory burden (30, 110). This is partly mediated through shared pathways like the Nrf2 signaling pathway, which, similar to exercise adaptation, can increase the capacity of the endogenous antioxidant system upon long-term polyphenol consumption (111, 112). Furthermore, both interventions influence gut microbiota modulation. Polyphenols significantly impact the composition of the gut microbiota, promoting beneficial microbes and inhibiting pathogenic ones, which contributes to improved metabolism and reduced inflammation (10, 113). While not explicitly detailed in the provided materials for exercise, it is widely recognized that physical activity also influences gut microbiota composition (114). This shared influence on gut health fosters a healthier metabolic environment, indirectly enhancing the overall anti-obesity effects. Finally, both polyphenols and exercise contribute to improved insulin sensitivity and glucose metabolism. Polyphenols target pathways such as PI3K/Akt and AMPK to enhance glucose uptake and utilization (19, 115). Concurrently, exercise is well-established for its ability to improve insulin sensitivity and glucose metabolism throughout the body (1, 116). The convergence of these actions provides a robust mechanism for addressing insulin resistance, a hallmark of obesity. The recurring themes of AMPK, SIRT1, and anti-inflammatory/antioxidant actions across both interventions are not coincidental; they represent critical points of convergence where polyphenols can amplify or fine-tune the effects of exercise, leading to a more comprehensive and effective metabolic re-programming (117, 118) (Figure 4).

## Clinical implications, current limitations, and future research directions

The collective evidence strongly suggests that the combined approach of polyphenol supplementation and regular exercise holds significant promise as a non-pharmacological strategy for obesity management. This multi-targeted intervention is particularly appealing for a complex disease like obesity, offering the potential to improve quality of life and prevent associated chronic metabolic disorders (78, 119). However, a significant portion of the mechanistic evidence, particularly for potent synergistic effects like WAT browning is derived from promising preclinical animal models, and the translation of these synergistic effects into consistent human outcomes faces several limitations. Human studies on the anti-obesity impact of polyphenols, both alone and in combination with exercise, are often inconsistent (5, 120). This variability can be attributed to a multitude of factors, including differences in study design and duration, variations among subjects (e.g., age, gender, ethnicity), the specific chemical forms of polyphenols used, and confounding lifestyle factors (5, 121). A significant challenge for polyphenols lies in their inherent instability, low solubility, rapid metabolism, poor absorption, and consequently, low oral bioavailability (19, 122). Beyond these biochemical hurdles, practical challenges in human interventions, such as ensuring long-term adherence to both exercise and supplementation protocols, remain a significant barrier to translation. The efficacy of these interventions is also deeply embedded within a broader lifestyle context, including overall dietary patterns, stress levels, and sleep quality, which can confound or modify the observed outcomes (5, 121). Currently, there is no consensus on a specific dosage or form of presentation that consistently yields the best results in human populations (13, 123). Furthermore, the observed synergy is not universal; some combinations may not yield additional benefits for certain physiological outcomes, as exemplified by the lack of additional benefit for cardiac hypertrophy when grape polyphenols were combined with exercise (84, 124). This suggests that effects can be physical-activity-status-specific, requiring tailored approaches. Moreover, some exercise-mediated changes in adipose tissue, such as those in the MAPK pathway, have been observed to be transient, losing significance days after the last exercise session, indicating a need for sustained intervention or consistent polyphenol support to maintain benefits (60, 125). The current research, while promising, highlights a critical translational gap between preclinical findings and consistent human outcomes. Overcoming this requires a shift towards more sophisticated clinical trial designs that account for the complex interplay of polyphenol bioavailability, exercise adherence, individual variability, and the specific molecular targets of synergy.

To bridge this translational gap and fully realize the clinical potential of this combined strategy, several future research directions are imperative. First, there is a critical need for more rigorous, well-designed, long-term randomized controlled trials in diverse human populations to bridge the translational gap from the promising animal data to human populations. These studies must aim to validate synergistic effects, establish optimal dosages, identify the most effective forms of polyphenols, and determine appropriate intervention durations (5, 126). Second, further mechanistic elucidation is required to precisely understand how polyphenols enhance or stabilize exercise-induced adaptations at the molecular and cellular levels, particularly concerning white adipose tissue browning and muscle anabolism (15, 127). Third, research should explore personalized approaches, taking into account individual genetic variations, gut microbiome profiles, and physical activity status to optimize polyphenol-exercise combinations for maximum efficacy (45). Fourth, the development of novel delivery systems is crucial. Strategies such as encapsulation in nanoparticles (e.g., liposomes, solid lipid nanoparticles) or formulation as phytosomes can protect these bioactive compounds from rapid gastrointestinal degradation, enhance their solubility, and improve transport across intestinal barriers (78, 128). Research must focus on these technologies to overcome the fundamental limitations of poor absorption and stability, which is essential for achieving consistent and therapeutic bioavailability in human trials (13, 129–131).

## Conclusion

Obesity stands as a formidable and escalating global health challenge, necessitating innovative and comprehensive intervention strategies. This review underscores that both dietary polyphenols and regular exercise are potent independent modalities in combating obesity and its associated metabolic derangements. Crucially, emerging scientific evidence increasingly points towards compelling synergistic effects when these two interventions are combined. This synergy is particularly evident in the promotion of white adipose tissue browning, a process that significantly enhances energy expenditure, and in the augmentation of muscle function and recovery, which are vital for sustained physical activity and metabolic health. These benefits are frequently mediated through the convergence of both polyphenols and exercise on key molecular pathways, such as the activation of AMP-activated protein kinase (AMPK) and the SIRT1-PGC-1α axis, alongside their shared capacity to exert robust antioxidant and anti-inflammatory responses. While the promise of this combined approach is substantial—particularly the compelling synergistic effects seen in preclinical models—the principal challenge remains bridging the translational gap. This gap is defined by the often-inconsistent results observed in human trials, which stem from unresolved issues related to effective dosage, poor bioavailability, and individual variability. The future of obesity management lies not in single magic bullets but in integrated, personalized strategies that leverage the multifaceted benefits of both lifestyle interventions and bioactive compounds. Continued rigorous research focusing on precise mechanistic elucidation, personalized protocols, and novel delivery systems will be instrumental in translating these promising preclinical findings into clinically validated, effective interventions, ultimately offering a more comprehensive and sustainable path towards combating obesity and improving global metabolic health.
